# Molecular and physiologic mechanisms of advanced ripening by trunk girdling at early veraison of ‘Summer Black’ grape

**DOI:** 10.3389/fpls.2022.1012741

**Published:** 2022-10-18

**Authors:** Yanjie Peng, Xingjia Gu, Qi Zhou, Jiao Huang, Zhong Liu, Yong Zhou, Ying Zheng

**Affiliations:** ^1^ College of Life Science, Leshan Normal University, Leshan, China; ^2^ Institution of Biodiversity Conservation and Utilization in Mount Emei, Leshan Normal University, Leshan, China; ^3^ Justices, Equity, Diversity, and Inclusion Department, California Association of Resource Conservation Districts, Folsom, CA, United States; ^4^ Academy of Mount Emei, Leshan Normal University, Leshan, China; ^5^ Research Institution of Industrial Crop, Leshan Academy of Agricultural Sciences, Leshan, China

**Keywords:** color turning, earlier maturation, girdling, RNA sequencing, sugar accumulation, *Vitis* L.

## Abstract

Although the effects of girdling on grape berry development have been widely studied, the underlying mechanisms are poorly understood, especially at the molecular level. This study investigated the effect of trunk girdling on grape (*Vitis* L.) berry maturation. Girdling was performed on 5-year-old ‘Summer Black’ grapevines at early veraison, and transcriptional and physiologic analyses were performed. Trunk girdling promoted sugar accumulation and color development in berries and accelerated berry ripening by 25 days. Genes related to sucrose cleavage and polysaccharide degradation were upregulated at the transcriptional level, which was associated with increased monosaccharide accumulation and berry softening. Anthocyanin biosynthesis and accumulation were also enhanced by trunk girdling through the upregulation of anthocyanin biosynthesis genes including phenylalanine ammonia-lyase and UDP-glucose:flavonoid 3-*O*-glucosyltransferase (*UFGT*). The increased expression of two *VvUFGT* genes was accompanied by the upregulation of *VvMYBA2* under girdling. The upregulation of genes involved in ethylene biosynthesis and hormone (abscisic acid and brassinosteroid) responses and downregulation of genes involved in indoleacetic acid biosynthesis and response may have also promoted berry ripening in the girdling group. A total of 120 differentially expressed transcription factor genes from 29 gene families including *MYB*, *ERF*, and *MYB*-related were identified in the girdling group, which may participate in the regulation of berry development and ripening. These results provide molecular-level insight into the positive effects of trunk girdling on berry development in grapes.

## Introduction

Trunk girdling is a traditional horticulture practice for regulating the growth and development of fruit trees (Binkley et al., 2006). Girdling has been shown to control vegetative growth, promote blooming and fruit development, increase fruit yield, and improve fruit quality and is widely applied in the cultivation of fruit trees such as apple, citrus, sweet cherry, and grape (Rivas et al., 2006; Michailidis et al., 2020; Pereira et al., 2020; Matsumoto et al., 2021; Ülker and Kamiloğlu, 2021).

Grape (*Vitis* L.) is one of the most important fruit crops worldwide owing to the popularity of grape wine and table grapes (Chen et al., 2019). Many studies have examined the effects of girdling on grapefruit. For example, Crupi et al. (2016) performed cane girdling on 8-year-old *Vitis vinifera* L. (cv. Early Red Seedless) and found that yield per vine was increased by 19.2% than control in two consecutive years on average. In ‘Crimson Seedless’ grapevines treated by trunk girdling and ethephon, color index of red grapes (CIRG) value, marketable product percentage, soluble solid content, acidity, maturity index, and total phenolic content all differed significantly compared with those of the control (İşçi et al., 2020). It was demonstrated that girdling may improve the quality of spine grape berries by increasing the content of anthocyanins, soluble sugars, vitamin C, and aroma compounds and decreasing titratable acid (TA) content (Zhu et al., 2022). The levels of plant hormones such as abscisic acid (ABA) and gibberellic acid (GA) were also altered by girdling, which was shown to play a critical role in regulating berry development (Tyagi et al., 2020).

The molecular mechanisms underlying the positive effect of trunk girdling on grape development are poorly understood. In spine grapes after girdling, the increase of expression of a few genes related to the biosynthesis of anthocyanins and aromatic compounds as well as sucrose transport was confirmed by quantitative real-time (qRT)-PCR during the berry veraison stage (Zhu et al., 2022); metabolomic and transcriptomic analyses found that abscisic acid and gibberellin contents were higher in fruitlets from girdled vines and that genes of the phenylpropanoid pathway were induced by girdling (Tyagi et al., 2020). Based on the above studies, we hypothesized that trunk girdling might promote the early maturation of grapes by altering the expressions of genes related to sugar accumulation, color turning, and hormone regulation. Thus, in the present study, we carried out a global gene expression analysis to investigate the effect of trunk girdling at early veraison on grape berry maturation.

## Materials and methods

### Plant materials and treatments

The present study was carried out in the research grapery of Leshan Normal University, Leshan, China (29°42′N, 103°38′E). The region has a warm and humid climate, with a mean yearly temperature of 16.5°C–18°C and more than 300 frost-free days and 1,290 mm of rainfall annually. Approximately 80% of the annual rainfall occurs in summer.

Experiments were performed using the early-maturing ‘Summer Black’ grape variety. Five-year-old *V. vinifera* ‘Summer Black’ grapevines, grafted on “SO4” (*Vitis berlandieri* × *Vitis riparia*), were planted in lines with a spacing of 4 × 0.7 m. All these grapevines were from the Zhichang Institution of Research on Grape in Shandong Province (China). The vines were grown in a rain shelter with a Y-shaped training system. Thirty uniform grapevines were then randomly selected and assigned to the girdling (G) or control (CK) group. At the start of the veraison, 45 days after full bloom (DAFB), trunk girdling was implemented at 30 cm from the ground in the G group. A full circle was cut at a depth of 4 mm (without penetrating the xylem) and a width of 5 mm. The same watering and fertilization strategies were applied to the G and CK plants. A drip irrigation system was used to keep soil moisture at 70%–80% of field capacity. Grape berry samples were collected on day 0 (G0 and CK0), day 10 (CK10 and G10), day 20 (G20 and CK20), day 30 (G30 and CK30), and day 40 (G40 and CK40) after girdling, immediately frozen in liquid nitrogen, and stored at −80°C until use. Two berries from the top, middle, and bottom of one bunch were collected, and two bunches were randomly sampled from each grapevine. Twelve bunches from six grapevines in two treatments (three grapevines per treatment) were sampled on every sampling day. Each analyzed replicate consisted of a well-mixed powder of six berries from the same bunch (two from the top, middle, and base of the bunch). To measure total soluble solid (TSS) and TA contents, six replicates of fresh berries from each treatment group were collected and mixed as described above. For RNA sequencing (RNA-seq), one replicate consisting of a well-mixed sample of two bunches from the same grapevine and three replicates per treatment on each day were used. All six replicates were used for the measurement of physiologic and biochemical parameters. For the rest berries of each sampled bunch, two were randomly selected for measurement of the Color Index for Red Grapes (CIRG) by an NR110 colorimeter (3nh, China), and five in G40 and CK40 were randomly selected for grain weight, longitudinal diameter, and equatorial diameter measurement.

### Analysis of physiologic and biochemical parameters

TSS content was measured using a pocket refractometer PAL-1 (ATAGO, Tokyo, Japan). The acidity was determined by titrating with 0.1 N of NaOH (pH 8.1). The soluble sugar content (SSC) was measured with the phenol-sulfuric acid method. Total phenolic and anthocyanin contents were measured with the Folin–Ciocalteu and pH differential methods, respectively (Leng et al., 2016). Enzymes were extracted with HEPES-NaOH buffer (pH 7.5) containing 50 mM of HEPES-NaOH (pH 7.5), 10 mM of MgCl_2_, 1 mM of EDTA, 0.1% (w/v) bovine serum albumin, 0.5% (w/v) crosslinked polyvinylpyrrolidone (PVPP), 2.5 mM of dithiothreitol, and 10 mM of vitamin C. The solution was then dialyzed at 4°C for 24 h. The dialysate was diluted 1/10 in diluted extraction buffer (containing no PVPP). Sucrose synthase (SUS) biosynthetic and cleavage activities and sucrose phosphate synthase (SPS) activity were measured as described by Liu et al. (2015), and soluble acid and neutral invertase activities were determined as described by Jiang et al. (2014). Enzymes activities were only evaluated on days 10, 20, 30, and 40 when significant differences in the appearance and inner quality of grape berries were observed between the G and CK groups.

### RNA extraction

The total RNA of three biological replicates from the two groups on days 10, 20, and 30 was extracted from the tissue powder using Plant RNA Purification Reagent (Invitrogen, Carlsbad, CA, USA) according the manufacturer’s instructions. DNase I (Takara, Otsu, Japan) was used to remove genomic DNA during RNA extraction. RNA quality and quantity were verified with a NanoDrop2000 spectrophotometer (Thermo Fisher Scientific, Waltham, MA, USA), and RNA integrity was verified by agarose gel electrophoresis with RNA integrity number (RIN) determined using an Agilent 2100 Nano Bioanalyzer (Agilent Technologies, Santa Clara, CA, USA). All RNA samples used to construct sequencing libraries were of high quality (OD260/280 = 1.8–2.2, OD260/230 ≥ 2.0, RIN ≥ 8, 28S:18S ≥ 1.0, >1 μg).

### Library preparation and RNA-seq

Total RNA (2 μg) was used to prepare an RNA-seq transcriptome library using the TruSeq RNA sample preparation kit (Illumina, San Diego, CA, USA). First, mRNA was isolated using oligo(dT) beads and fragmented in a fragmentation buffer. The SuperScript double-stranded cDNA synthesis kit (Invitrogen) with random hexamer primers (Illumina) was then used to synthesize double-stranded cDNA, which was subjected to end repair, phosphorylation, and “A” base addition according to Illumina’s protocol for library construction. Target cDNA fragments with a length of 300 bp were selected on 2% low-range ultra agarose and PCR-amplified using Phusion DNA polymerase (New England Biolabs, Ipswich, MA, USA) over 15 cycles. The paired-end sequencing library was quantified with TBS380 (Picogreen, Invitrogen) and sequenced on a HiSeq PE150 sequencer (Illumina; 2 × 150-bp read length).

### Read mapping

The raw paired-end reads were trimmed and quality controlled to obtain clean reads using the default parameters of SeqPrep (https://github.com/jstjohn/SeqPrep) and Sickle (https://github.com/najoshi/sickle). Clean read alignment was then performed using HISAT2 software (Kim et al., 2015). The mapped reads were assembled with StringTie using a reference-based approach (Pertea et al., 2015). The *V. vinifera* genome was used as the reference genome for read mapping (12×; http://genomes.cribi.unipd.it/DATA/V2/V2/).

### Differential expression analysis and functional enrichment

Data were analyzed on the Majorbio Cloud online platform (www.majorbio.com). Gene expression levels were calculated as clean read counts and are presented as transcripts per million reads (TPMs). Gene abundance was qualified by RNA-seq and expectation maximization (Li and Dewey, 2011). DESeq2 was used to perform differential expression analysis (Love et al., 2014). Genes from samples in the G group with adjusted p-value (p_adj_) <0.05 and |log_2_ fold change| > 1 relative to the CK group were considered as differentially expressed genes (DEGs) with statistical significance. Gene Ontology (GO) and Kyoto Encyclopedia of Genes and Genomes (KEGG) pathway analyses were carried out using Goatools and KOBAS (Xie et al., 2011). GO terms and KEGG pathways with a Bonferroni-corrected p-value ≤0.05 were considered enriched.

### qRT-PCR validation of RNA-seq data

A total of 12 DEGs associated with berry development were selected for qRT-PCR validation of RNA-seq gene expression data (**Supplementary Table 1**). The same RNA used in RNA-seq was reverse transcribed using MonScript RTIII Super Mix with dsDNase (Monad, Wuhan, China). qRT-PCR was performed for three biologic replicates and three technical replicates using MonAmp ChemoHS qPCR Mix (Monad) on a qTOWER 3 Real-Time PCR Thermal Cycler (Analytik Jena, Jena, Germany). The primers used in this study were selected from the literature or designed using Premier 5 (Premier Biosoft, Palo Alto, CA, USA). Glyceraldehyde 3-phosphate dehydrogenase (*GAPDH*) was used as an internal control to normalize all data, and relative expression levels were calculated with the 2^−ΔΔCT^ method (Livak and Schmittgen, 2001).

### Statistical analysis

Results are shown as mean ± SE of at least three biological replicates. Differences between groups were evaluated by analysis of variance and with Student’s t-test using SPSS Statistics 25 (IBM, Armonk, NY, USA). Figures were produced using OriginPro 2016 (OriginLab, Northampton, MA, USA). p < 0.05 was considered statistically significant.

## Results

### Grape development

**Figure 1:**
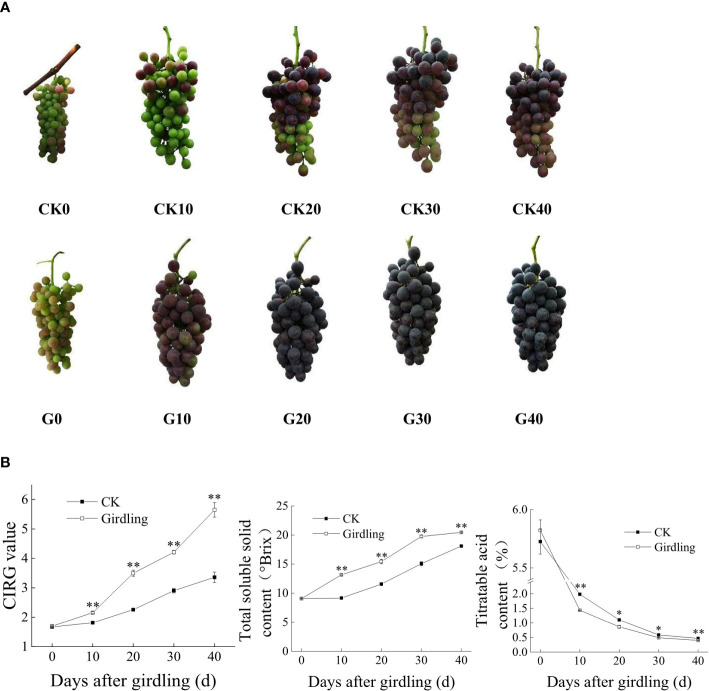
Berry development of ‘Summer Black’ after girdling treatment. **(A)** Bunches of the girdling and control groups on days 0, 10, 20, 30, and 40 after treatment. **(B)** CIRG value and TSS and TA contents of berries at each time point. *p < 0.05, **p < 0.001 *vs.* control on the same day (t-test). CIRG, color index of red grapes; TSS, total soluble solid; TA, titratable acid.

Girdling treatment accelerated the ripening of ‘Summer Black’ grape berries (**Figure 1**). G30 berries were fully ripe in appearance, whereas CK40 berries were still in the process of coloration, and the color of G20 berries was darker than that of CK40 berries (**Figure 1A**). Similar trends were observed for the CIRG value and TSS and TA contents of berries in both the G and CK groups, with significant differences in content between groups at any given time point after girdling (**Figure 1B**). TSS content increased whereas TA content decreased with time since girdling. In G40, the CIRG value was 5.65, TSS content was 20.47°Brix, TA content was 0.40%, and the solid-to-acid ratio was 51.2:1; in CK40 berries, the values were 3.36, 18.13°Brix, 0.47%, and 38.6:1, respectively. Overall, trunk girdling at early veraison promoted the development of ‘Summer Black’ berries in both appearance and physiologic properties. Taking the total TSS content >18°Brix and full red coloration as the standards of maturity, berries were already mature in G30 (75 DAFB), whereas CK30 berries matured at approximately 100 DAFB (data not shown). Thus, trunk girdling accelerated berry ripening by approximately 25 days. Additionally, the average grain weight, longitudinal diameter, and equatorial diameter of G40 berries were 8.423 g, 26.00 mm, and 23.15 mm, respectively, and in CK40, those values were 8.534 g, 26.07 mm, and 23.55 mm, respectively. There was no significant difference between G40 and CK40 in average berry grain weight and size.

### Evaluation of RNA-seq data

The features of all 18 libraries are summarized in **Supplementary Table 2**. RNA-seq generated an average of 8.37 Gb of raw data with 7.28–9.25 Gb clean bases. The Q30 scores of clean bases were all >92.43%. Each sample was represented by over 49.38 clean reads, which was sufficient for quantitative analysis of gene expression. For all samples, >86.72% of clean reads were matched to the grape reference genome.

### Principal component analysis, gene expression analysis, and qRT-PCR validation

**Figure 2:**
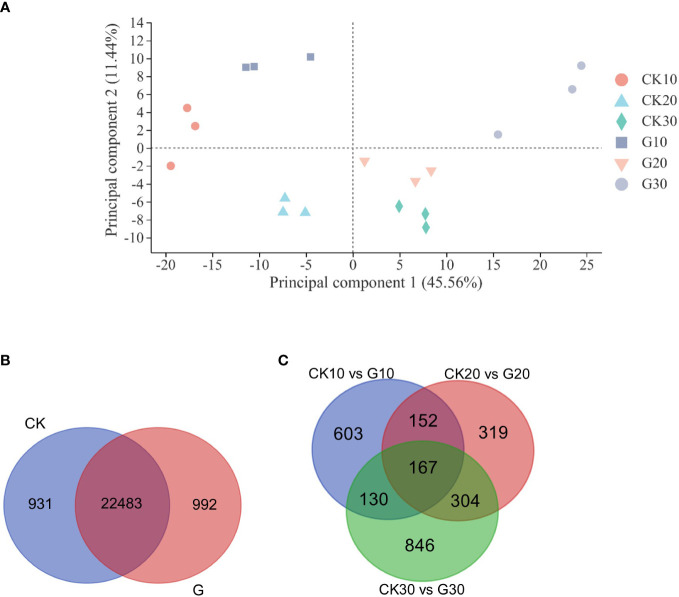
Principal component analysis and gene expression. **(A)** Principal component analysis of 18 RNA-seq libraries. TPM values were used in the analysis. **(B)** Venn diagram of expressed genes in girdling and control groups. **(C)** Venn diagram of DEGs between girdling and control groups at three different sampling times. TPM, transcript per million reads; DEGs, differentially expressed genes.

**Figure 3:**
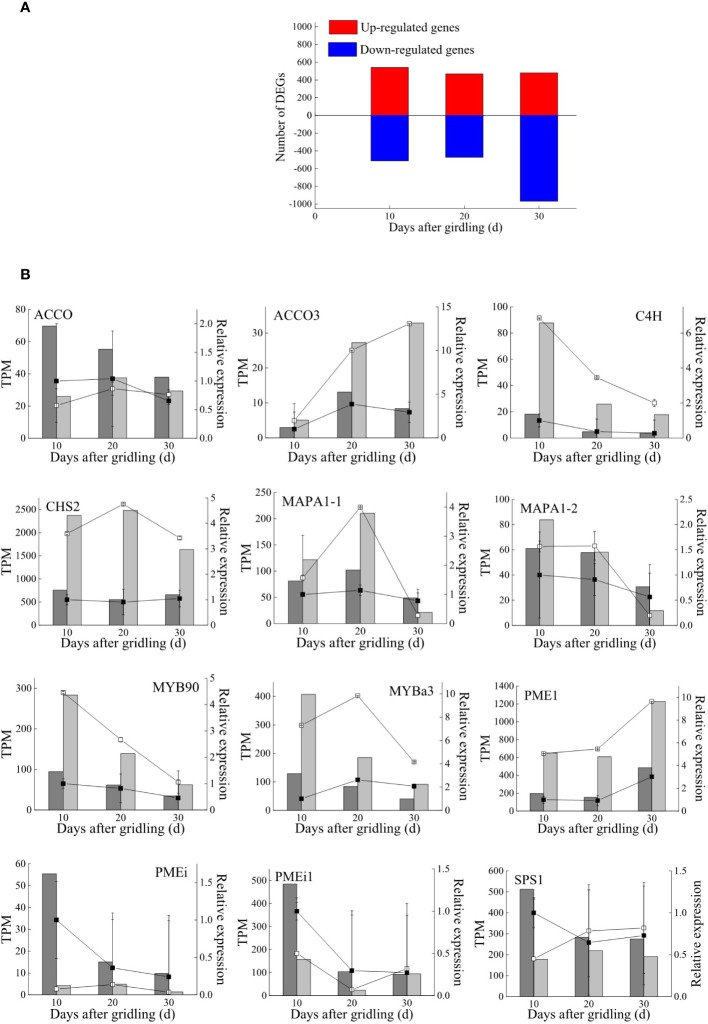
DEGs and qRT-PCR validation. **(A)** Number of DEGs between treatments at three sampling times. **(B)** Transcriptome validation by qRT-PCR. Gene expression levels of the girdling group (light bars) and control group (dark bars) obtained by RNA-seq are presented as TPM values; gene expression levels in the girdling group (empty squares) and control group (solid squares) obtained by qRT-PCR were normalized to those of glyceraldehyde 3-phosphate dehydrogenase (*GADPH*) expression. ACCO, amicyclopropanecarboxylate oxidase; C4H, trans-cinnamate 4-monooxygenase; CHS, chalcone synthase; MAPA, major allergen Pru ar; MYB, transcription factor MYB; PME, pectinesterase; PMEi, plant invertase/pectin methylesterase inhibitor; SPS, sucrose phosphate synthase.

Global gene expression levels differed between G and CK groups and between different sampling time points (**Figure 2A**). There were 24,406 genes expressed in at least one of the 18 libraries, including 992 and 931 genes expressed only in the G and CK groups, respectively (**Figure 2B** and **Supplementary Table 3**). A total of 2,521 DEGs were identified between the G and CK groups; 1,052 genes were expressed differently on day 10, 942 on day 20, and 1,447 on day 30 in the G group; 603, 319, and 846 genes were only expressed differently on days 10, 20, and 30, respectively (**Figure 2C** and **Supplementary Table 4**). The number of upregulated and downregulated DEGs was similar on days 10 and 20; however, there were 967 DEGs that were downregulated and 480 DEGs that were upregulated on day 30 (**Figure 3A**). To validate the accuracy of gene expression data obtained by RNA-seq, 12 DEGs involved in grape development were selected for qRT-PCR analysis. The relative expression of the 12 DEGs was in line with the expression levels determined by RNA-seq (**Figure 3B**), confirming the accuracy and reliability of the RNA-seq profiles.

### Functional annotation and classification of differentially expressed genes

**Figure 4:**
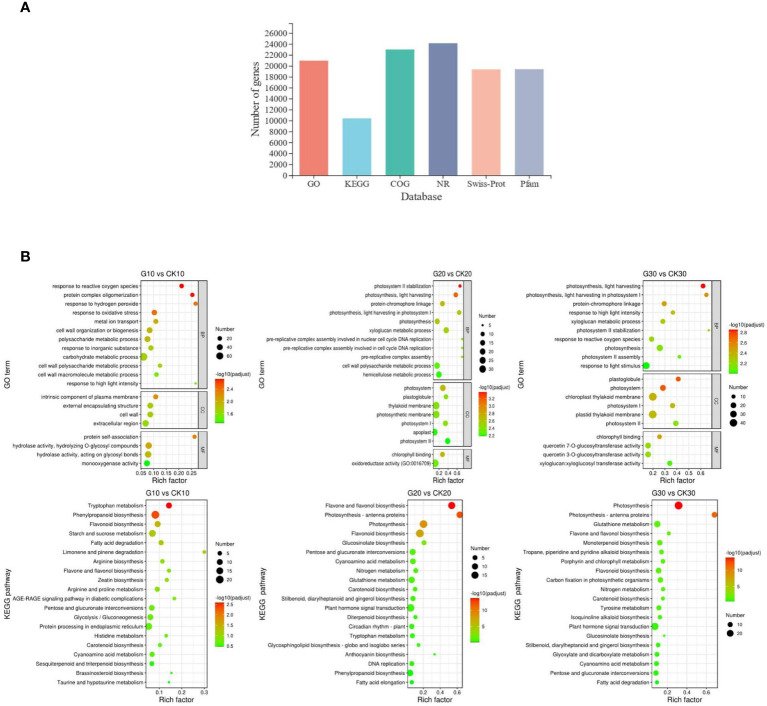
Functional annotation and DEG enrichment analysis. **(A)** Functional annotation of DEGs against six public databases. **(B)** Top 20 enriched GO terms and KEGG pathways in girdling vs. control group. GO terms were classified into three categories: biological process (BP), cellular component (CC), and molecular function (MF). DEG, differentially expressed gene; GO, Gene Ontology; KEGG, Kyoto Encyclopedia of Genes and Genomes.

The 24,406 expressed genes were annotated using six public databases; 85.81% were annotated in GO, 42.63% in KEGG, 94.19% in Evolutionary Genealogy of Genes: Non-supervised Orthologous Groups (EggNOG), 98.87% in National Center for Biotechnology Information Nonredundant Protein (NR), 79.28% in Swiss-Prot, and 79.41% by Protein Family (Pfam) databases (**Figure 4A** and **Supplementary Table 5**).

The DEGs were significantly enriched in 20, 46, and 36 GO terms in samples from days 10, 20, and 30, respectively; the top 20 enriched GO terms are shown in **Figure 4B**. The DEGs from day 10 were mainly related to the response to stress, as three of the top 4 enriched biological processes were associated with oxidative stress; at later time points, the most enriched GO terms were mainly related to photosynthesis.

The DEGs were significantly enriched in 3, 5, and 10 KEGG pathways in samples from days 10, 20, and 30, respectively (**Figure 4B**). The pathways were mainly associated with anthocyanin biosynthesis including phenylpropanoid, flavonoid, and flavone/flavonol biosynthesis pathways. Several significantly enriched pathways were also associated with photosynthesis such as the photosynthesis and photosynthesis-antenna protein pathways, consistent with the terms identified by GO analysis.

### Effect of girdling on the regulation of fruit color change

**Figure 5:**
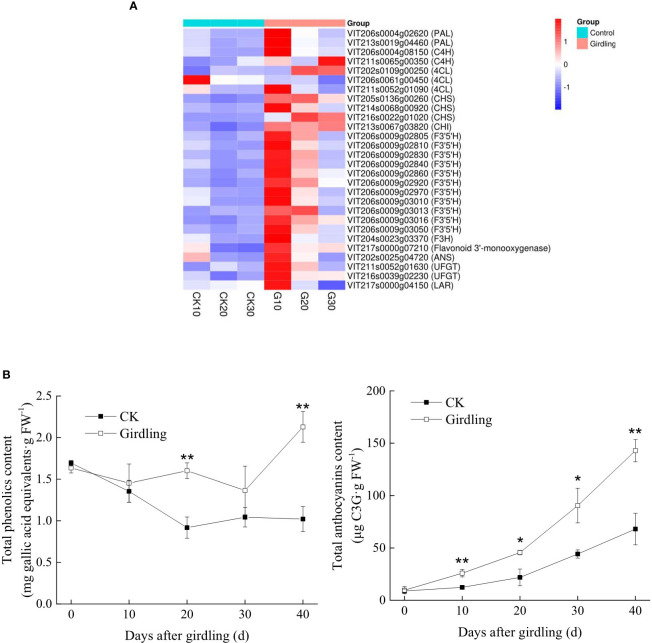
Effects of girdling treatment on fruit color change in ‘Summer Black’. **(A)** Heatmap of DEGs related to anthocyanin biosynthesis. TPMs of genes were used in the analysis after row z-score scaling. DEGs are labeled by gene ID (description). 4CL, 4-coumarate coenzyme A ligase; ANS, anthocyanidin synthase; CHI, chalcone isomerase, CHS, chalcone synthase; F3′5′H, flavonoid 3′,5′-hydroxylase; F3H, naringenin 3-dioxygenase; LAR, leucoanthocyanidin reductase; PAL, phenylalanine ammonia lyase; UFGT, UDP-glucose:flavonoid 3-*O*-glucosyltransferase. **(B)** Total phenolic and anthocyanin contents. *p < 0.05 and ** p < 0.01 *vs.* control on the same day (t-test). DEGs, differentially expressed genes; TPMs, transcripts per million reads.

Trunk girdling at early veraison caused differential expression of 28 genes *vs.* the CK group on at least one sampling day, including genes in the phenylpropanoid, flavonoid, and anthocyanin biosynthesis pathways, most of which were upregulated (**Figure 5A**). These 28 genes were all related to anthocyanin biosynthesis, which plays an important role in the color change of grape berries. Among the 28 genes, 20, 4, and 1 were most active in G10, G20, and G30, respectively, suggesting that girdling promoted anthocyanin biosynthesis in grape berries. Only two genes were significantly downregulated relative to the control. One of these was VIT206s0061g00450 (4-coumarate coenzyme A ligase [4CL] KEGG Orthology [KO] group), whose transcript abundance in G10 was only 49.3% of that in CK10. However, VIT211s0052g01090 and VIT202s0109g00250 (also in KO 4CL) were significantly upregulated. The other significantly downregulated gene was VIT217s0000g04150, which is in the leucoanthocyanidin reductase (LAR) KO group. LAR is an enzyme that generates intermediate products for the biosynthesis of other phenolics. The transcript abundance of VIT217s0000g04150 in G30 was only 44.2% of that in CK30.

Compared with the CK group, total phenolic and anthocyanin contents were increased by girdling (**Figure 5B**), in line with the results of the transcriptome analysis. Total phenolic content was significantly higher in G20 and G40 than in corresponding CK groups (p < 0.05); total anthocyanin content was significantly higher in the girdling groups than in the corresponding CK groups after girdling (p < 0.05): on days 10, 20, 30, and 40, total anthocyanin content was 2.10, 2.09, 2.05, and 2.10 times higher than in the corresponding CK groups, providing further evidence that the fruit color of berries was markedly altered by girdling.

### Effect of girdling on regulation of berry photosynthesis

**Figure 6:**
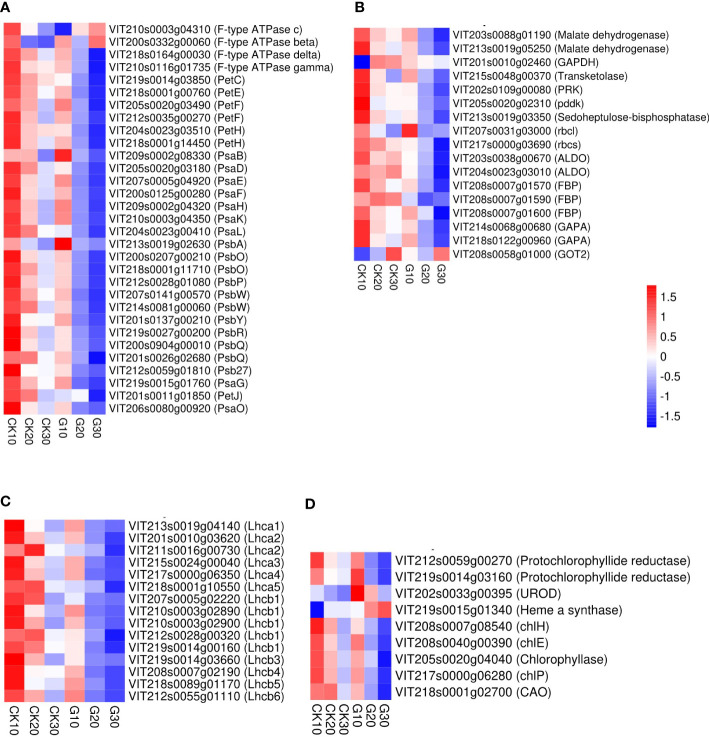
Heatmaps of DEGs related to photosynthesis of grape berries. **(A)** DEGs in the photosynthesis pathway. Pet, photosynthetic electron transport; Psa, photosystem I subunit; Psb, photosystem II subunit; DEGs, differentially expressed genes. **(B)** DEGs in the photosynthesis-antenna proteins pathway. ALDO, fructose-bisphosphate aldolase; FBP, fructose-1,6-bisphosphatase; GAPA, glyceraldehyde-3-phosphate dehydrogenase; GOT2, aspartate aminotransferase; pddk, pyruvic-phosphate dikinase; PRK, phosphoribulokinase; rbcl, ribulose-bisphosphate carboxylase large chain; rbcs, ribulose-bisphosphate carboxylase small chain. **(C)** DEGs in the carbon fixation in photosynthetic organisms pathway. Lhca, light-harvesting complex I chlorophyll a/b binding protein; Lhcb, light-harvesting complex II chlorophyll a/b binding protein. **(D)** DEGs in the porphyrin and chlorophyll metabolism pathway. CAO, chlorophyllide a oxygenase chlE, magnesium-protoporphyrin IX monomethyl ester (oxidative) cyclase; chlH, magnesium chelatase subunit H; chlP, geranylgeranyl diphosphate/geranylgeranyl-bacteriochlorophyllide a reductase; UROD, uroporphyrinogen decarboxylase. TPMs of genes were used in the analysis after row z-score scaling. DEGs were labeled by gene ID (description). A color scale is shown in all four heatmaps.

Girdling led to the downregulation of berry photosynthesis-related genes compared with the CK group. Among the 72 DEGs were genes in the Photosynthesis (**Figure 6A**), Photosynthesis antenna protein (**Figure 6B**), Carbon fixation in photosynthetic organism (**Figure 6C**), and Porphyrin and chlorophyll metabolism (**Figure 6D**) KEGG pathways. These four pathways play critical roles in plant photosynthesis. Only two DEGs were downregulated in G10, whereas 35 and 59 were downregulated in G20 and G30, respectively. Meanwhile, four, one, and two DEGs were upregulated in G10, G20, and G30, respectively (**Figure 6** and **Supplementary Table 4**). Over time, the expression of photosynthesis-related genes decreased in both groups. The lowest transcript levels of most DEGs were observed on day 30; the exceptions were VIT210s0003g04310, VIT200s0332g00060, VIT208s0058g01000, and VIT219s0015g01340.

### Effect of girdling on polysaccharide degradation and disaccharide metabolism

**Figure 7:**
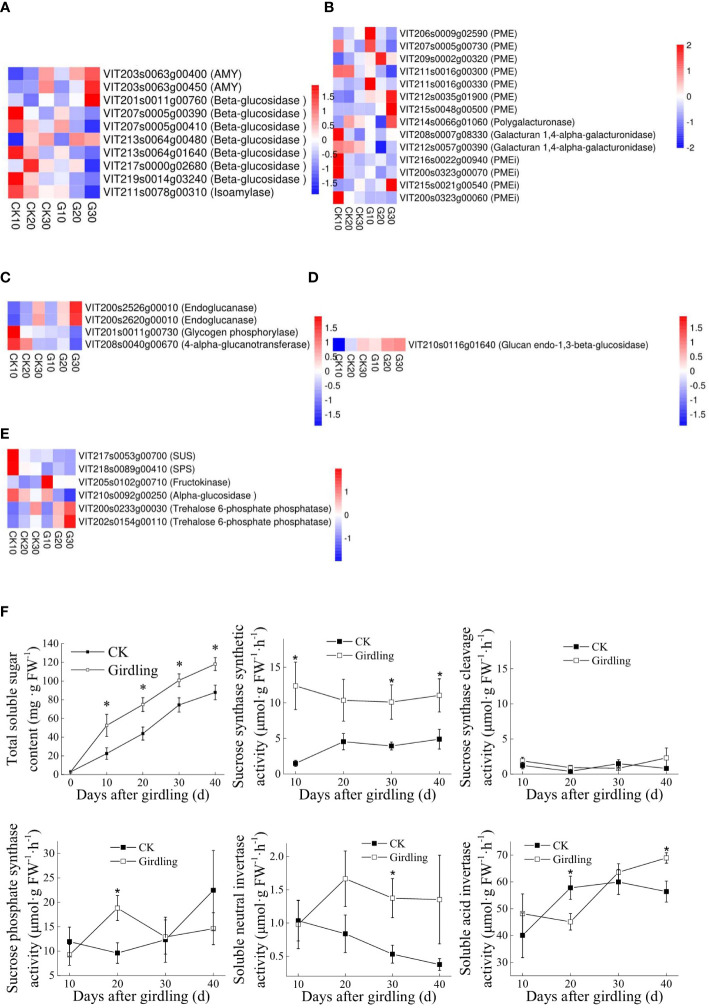
Effects of girdling on polysaccharide degradation and disaccharide metabolism. **(A–F)** Expression patterns of DEGs associated with starch degradation **(A)**, pectin degradation **(B)**, cellulose degradation **(C)**, 3-beta-d-glucan hydrolysis **(D)**, and disaccharide metabolism **(E)** are as heatmaps. TPMs of genes were used in the analysis after row z-score scaling. DEGs are labeled by gene ID (description). AMY, alpha-amylase; SUS, sucrose synthase; DEGs, differentially expressed genes; TPMs, transcripts per million reads. **(F)** Total SSC and activity of enzymes participating in sucrose metabolism. SSC, soluble sugar content. *p < 0.05 *vs.* control on the same day (t-test).

Girdling increased polysaccharide degradation at the level of transcription. The expression level of several genes related to the degradation of major polysaccharides was upregulated under girdling, including two alpha-amylase (AMY) genes (VIT203s0063g00400 and VIT203s0063g00450; **Figure 7A**), two endoglucanase genes (VIT200s2526g00010 and VIT200s2620g00010; **Figure 7C**), and one glucan endo-1,3-β-glucosidase gene (VIT210s0116g01640; **Figure 7D**). There were six pectin esterase (PME) genes among the DEGs, five of which were upregulated under girdling; VIT211s0016g00300 was the only gene that was downregulated (**Figure 7B**). One invertase pectin methylesterase inhibitor (PEMi) gene (VIT215s0021g00540) was upregulated, and three PEMi genes were downregulated (**Figure 7B**), indicating that there was less inhibition of PME activity and that pectin degradation was enhanced under girdling. Total SSC was significantly higher in the G group than in the CK group (**Figure 7F**), consistent with the observed changes in transcript levels of DEGs related to polysaccharide degradation.

There were six DEGs related to disaccharide metabolism (**Figure 7E**). VIT217s0053g00700 and VIT218s0089g00410, encoding SUS and SPS, were downregulated under girdling, suggesting that sucrose biosynthesis from glucose and fructose was suppressed.

The activity of enzymes involved in sucrose metabolism was also influenced by girdling treatment (**Figure 7F**). Sucrose biosynthesis was more active than sucrose cleavage under girdling, especially in the G group compared with the CK group. SPS activity was also significantly higher in the G group on day 20, despite the slight downregulation of VIT218s0089g00410 at this time point. Although there was no difference in invertase gene expression between groups, the activity of invertases differed: soluble neutral invertase had higher activity in the G group than in the CK group on day 30, although it plays a minor role in sucrose degradation (**Figure 7F**); meanwhile, soluble acid invertase, which is important for sucrose cleavage, had significantly lower activity on day 20 and higher activity on day 40 in the G group than in the CK group.

### Effect of girdling on hormone biosynthesis and response

Girdling affected hormone biosynthesis, as evidenced by the 23 DEGs associated with seven hormone biosynthesis processes (**Table 1**). In general, the biosynthesis of indoleacetic acid (IAA), cytokinin (CK), gibberellin (GA), ABA, and brassinosteroid (BR) was negatively influenced by girdling: six of the seven genes involved in ABA biosynthesis (except VIT205s0051g00670 [NCED]) showed lower expression in the G group than in the CK group. VIT218s0001g11630 (AOS) and VIT200s0299g00010 (ACOX), both involved in JA biosynthesis, were upregulated and downregulated, respectively, by girdling. Expression levels of the ethylene (ETH) biosynthesis genes VIT208s0007g04990 (MelK), VIT208s0007g05000 (MelK), and VIT212s0059g01380 (ACCO) were higher whereas the levels of VIT202s0025g00360 (ACS) and VIT200s2086g00010 (ACCO) were lower in the G group than in the CK group. Additionally, VIT211s0016g02380 (ACCO) was upregulated on day 10 but downregulated on day 20 by girdling.

**Table 1:** Changes in the expression of DEGs related to plant hormone biosynthesis.

Gene ID	Description	Fold change in TPM		
		Day 10	Day 20	Day 30
**IAA biosynthesis**				
VIT207s0104g01250	Indole-3-pyruvate monooxygenase (YUCCA)	0.48*	0.41*	0.4*
**CK biosynthesis**				
VIT207s0104g00270	Cytokinin synthase (IPT)	0.33*	0.99	1.06
**GA biosynthesis**				
VIT218s0001g11320	*ent*-Kaurene oxidase (GA3)	0.35*	0.38*	0.41*
VIT219s0140g00140	Gibberellin 2 beta-dioxygenase (GA2ox)	0.42	0.43*	0.49
**ABA biosynthesis**				
VIT216s0050g01090	Beta-carotene 3-hydroxylase (CrtZ)	0.81	0.4*	0.26*
VIT207s0031g00620	Zeaxanthin epoxidase (ZEP)	0.55	0.51	0.26*
VIT202s0087g00910	9-*cis*-Epoxycarotenoid dioxygenase (NCED)	0.32*	1.44	0.58
VIT202s0087g00930	9-*cis*-Epoxycarotenoid dioxygenase (NCED)	0.35	0.19*	0.89
VIT205s0051g00670	9-*cis*-Epoxycarotenoid dioxygenase (NCED)	6.06*	2.96	2.25
VIT210s0003g03750	9-*cis*-Epoxycarotenoid dioxygenase (NCED)	0.11*	0.31*	1.3
VIT218s0041g02410	Indole-3-acetaldehyde oxidase (AAO1)	0.52	0.2*	0.21*
**BR biosynthesis**				
VIT208s0007g01760	Steroid 5-alpha-reductase (DET2)	0.59	0.8	0.21*
VIT204s0023g02650	3-Epi-6-deoxocathasterone 23-monooxygenase (CYP90C1)	0.73	0.27*	0.14
VIT214s0083g01110	Brassinosteroid-6-oxidase 1 (CYP85A1)	0.3*	0.6	0.86
VIT211s0016g04810	PHYB activation tagged suppressor 1 (CYP734A1)	0.26*	0.4	0.5
**JA biosynthesis**				
VIT218s0001g11630	Hydroperoxide dehydratase (AOS)	1.6	1.62	2.98*
VIT200s0299g00010	Acyl-CoA oxidase (ACOX)	0.77	0.92	0.41*
**ETH biosynthesis**				
VIT208s0007g04990	*S*-Adenosylmethionine synthetase (MetK)	1.79	1.7	3.12*
VIT208s0007g05000	*S*-Adenosylmethionine synthetase (MetK)	7.66*	5.98*	5.38*
VIT202s0025g00360	1-Aminocyclopropane-1-carboxylate synthase (ACS)	0.68	0.5*	0.63
VIT200s2086g00010	ACCO	0.39*	0.69	0.9
VIT211s0016g02380	ACCO	7.58	0.28*	1.24
VIT212s0059g01380	ACCO	1.74	2.16*	4.65*

Fold changes in TPM were calculated as the ratio of TPM in the girdling group relative to the control group.

ABA, abscisic acid; BR, brassinosteroid; CK, cytokinin; ETH, ethylene; GA, gibberellic acid; IAA, indoleacetic acid.

*p_adj_ < 0.05 vs. control on the same day.

There were 34 DEGs related to hormone responses (**Table 2**), including 15 related to IAA response; three IAA, one ARF, two GH3, and six SAUR genes were downregulated by girdling whereas only VIT203s0038g02140 (AUX1), VIT213s0067g00330 (AUX1), and VIT205s0020g01070 (IAA) were significantly upregulated on day 20. Unlike the expressing patterns of hormone biosynthesis genes, most DEGs related to CK, ABA, and BR responses were upregulated in the G group relative to the CK group.

**Table 2:** Changes in the expression of DEGs related to plant hormone response.

Gene ID	Description	Fold change in TPM		
		Day 10	Day 20	Day 30
**IAA response**				
VIT203s0038g02140	Auxin influx carrier (AUX1)	0.34	4.59*	2.13
VIT213s0067g00330	Auxin influx carrier (AUX1)	0.65	2.15*	1.85
VIT205s0020g01070	Auxin-responsive protein IAA (IAA)	1	5.86*	0.23*
VIT207s0141g00290	Auxin-responsive protein IAA (IAA)	0.48*	0.81	0.78
VIT209s0002g04080	Auxin-responsive protein IAA (IAA)	0.16*	0.38*	0.34
VIT211s0016g03540	Auxin-responsive protein IAA (IAA)	0.55	1.06	0.34*
VIT202s0025g01740	Auxin response factor (ARF)	0.67	0.53	0.38*
VIT203s0091g00310	Auxin-responsive GH3 gene family (GH3)	0.68	0.76	0.38*
VIT207s0005g00090	Auxin-responsive GH3 gene family (GH3)	0.15*	0.48	0.4
VIT203s0038g00940	SAUR family protein (SAUR)	0.48	0.87	0.26*
VIT203s0038g01130	SAUR family protein (SAUR)	0.71	0.51	0.15*
VIT203s0038g01285	SAUR family protein (SAUR)	0.54	0.29*	0.06
VIT204s0023g00530	SAUR family protein (SAUR)	0.89	2.15	0.21*
VIT207s0031g02740	SAUR family protein (SAUR)	0.25*	1.84	1.89
VIT216s0098g01150	SAUR family protein (SAUR)	0.28*	1.72	1.66
**CK response**				
VIT201s0011g04220	ARR-B family (ARR-B)	4.18*	0.36*	1
VIT201s0026g00940	ARR-A family (ARR-A)	1.41	1.65	2.31*
VIT213s0067g03070	ARR-A family (ARR-A)	3.09*	1.32	1.9
**GA response**				
VIT207s0005g05100	Phytochrome-interacting factor 4 (PIF4)	0.71	0.51	0.3*
**ABA response**				
VIT216s0050g02680	Protein phosphatase 2C (PP2C)	9.1*	2.64*	43.59*
**BR response**				
VIT211s0052g01180	Xyloglucosyl transferase TCH4 (TCH4)	1.5	26.92*	4.66*
VIT211s0052g01190	Xyloglucosyl transferase TCH4 (TCH4)	0.83	72.81*	3.71*
VIT211s0052g01200	Xyloglucosyl transferase TCH4 (TCH4)	9.09	76.52*	2.94
VIT211s0052g01220	Xyloglucosyl transferase TCH4 (TCH4)	5.14	8.52	13.35*
VIT211s0052g01250	Xyloglucosyl transferase TCH4 (TCH4)	8.26*	3.26*	1.2
VIT211s0052g01260	Xyloglucosyl transferase TCH4 (TCH4)	3.31*	22.65*	2.59*
VIT211s0052g01270	Xyloglucosyl transferase TCH4 (TCH4)	2.77	77.17*	4.44*
VIT211s0052g01280	Xyloglucosyl transferase TCH4 (TCH4)	1	79.76*	12*
VIT211s0052g01300	Xyloglucosyl transferase TCH4 (TCH4)	0.9	41.11*	3.9*
VIT211s0052g01320	Xyloglucosyl transferase TCH4 (TCH4)	1	35.44	92.1*
VIT211s0052g01330	Xyloglucosyl transferase TCH4 (TCH4)	0.32	72.96*	4.38*
VIT211s0052g01340	Xyloglucosyl transferase TCH4 (TCH4)	2.06	93.18*	5.37*
VIT203s0180g00040	Cyclin D3, plant (CYCD3)	0.47*	0.89	0.58
VIT218s0001g01240	Cyclin D3, plant (CYCD3)	0.7	0.49	0.12*

Fold changes in TPM were calculated as the ratio of TPM in the girdling group relative to the control group.

ABA, abscisic acid; BR, brassinosteroid; CK, cytokinin; GA, gibberellic acid; IAA, indoleacetic acid.

*p_adj_ < 0.05 vs. control on the same day.

### Expression of differentially expressed genes encoding transcription factors under girdling treatment

**Figure 8:**
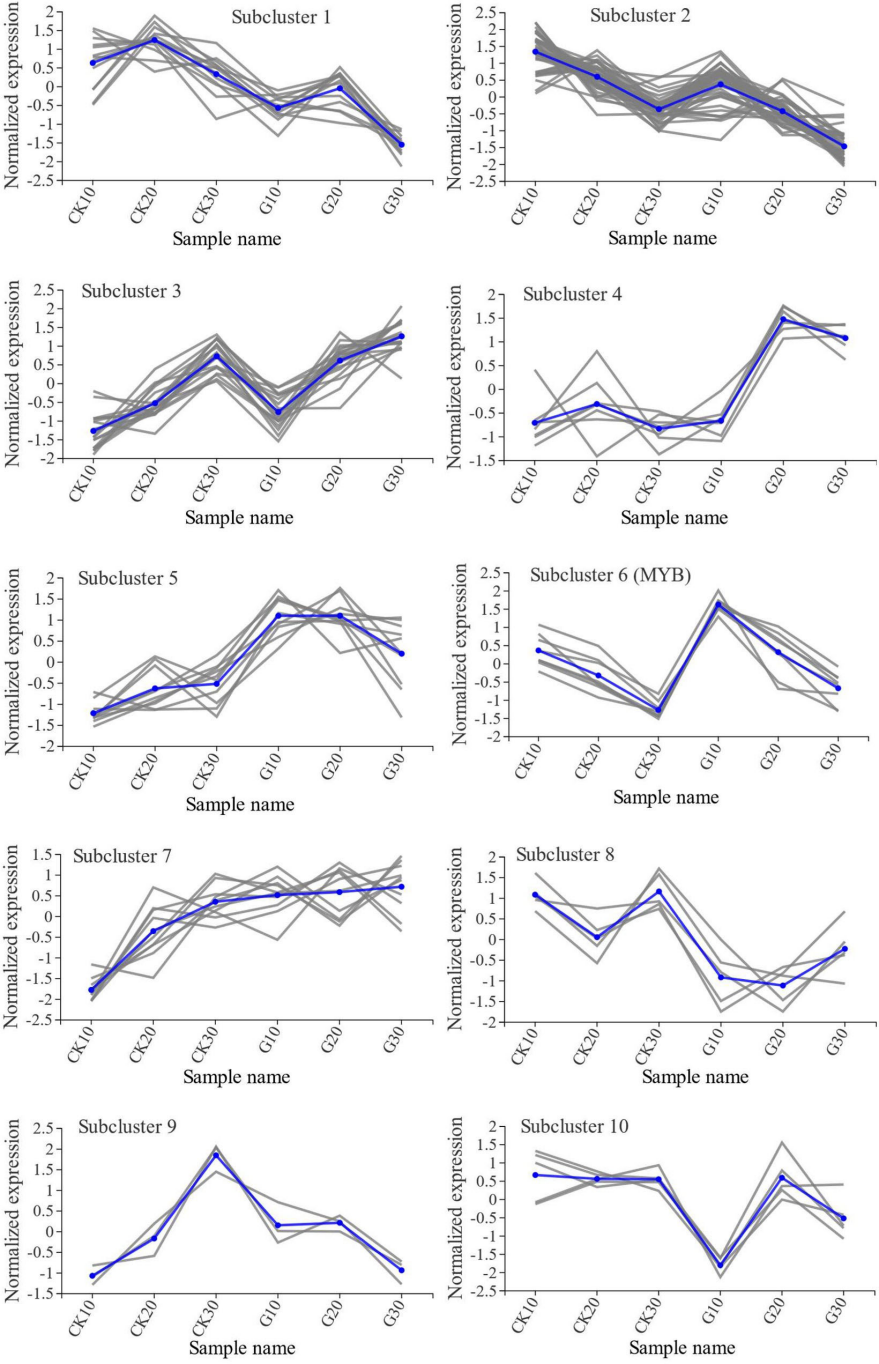
Cluster analysis of DEGs encoding transcription factors. DEGs were classified into 10 subclusters by K-means cluster analysis. Blue lines represent the average trends of all gene expressions within subclusters.

Compared with the CK group, girdling caused the differential expression of 120 transcription factor genes from 29 gene families (**Supplementary Table 6**). There were 57, 52, and 61 DEGs on days 10, 20, and 30, respectively. The three most highly represented families were ERF-, MYB-, and MYB-related with 14, 13, and 12 DEGs, respectively. Among the differentially expressed transcription factor genes, the MYB family gene VIT202s0033g00390 (VvMYBA2) and GATA family gene VIT204s0008g01290 showed the largest differences in expression relative to the CK group (21.7- and 0.037-fold, respectively, of the CK group). The 120 DEGs were classified into 10 subclusters by K-means cluster analysis (**Figure 8**). Subclusters 2 and 3 had the most differentially expressed transcription factor genes with 40 and 19, respectively; in these subclusters, the change in gene expression over time and the level of expression were similar between the G and CK groups. In subclusters 1, 6, and 8, the change in gene expression over time was also similar between treatment groups, whereas expression levels differed significantly. Divergent gene expression curves between G and CK groups were also observed in subclusters 4, 5, 7, 9, and 10, suggesting that girdling had a greater effect on the expression of genes in these subclusters than on other transcription factor-encoding genes.

## Discussion

In grapevines, girdling is typically performed either after fruit set or at early veraison; the former increases berry size, whereas the latter improves fruit color and promotes fruit maturation (Tyagi et al., 2020). In our study, trunk girdling at early veraison altered the physiologic characteristics and appearance of grape berries (**Figure 1**), leading to earlier ripening compared with the control. Phyto-technical methods like girdling were widely applied in both table grape and wine grape cultivation to produce berries with high quality, as well as to avoid potential climate change impacts (Neumann and Matzarakis, 2014; Tóth, 2020).

High TSS and low TA contents lead to a high solid-to-acid ratio, which is widely used as the physiologic standard for the maturity of grape berries. Soluble sugars are the main contributor to soluble solids in grape berries; their rapid accumulation is a critical process in the veraison and ripening stages. Most sugars in fruits are derived from photosynthates, which are produced by leaves and transferred in the form of sucrose to developing berries and other organs through the phloem (Swanson and El-Shishiny, 1958; Koch, 1984). After trunk girdling, little or no photosynthates were transferred to the lower part of the wound; instead, the photosynthates were partly reallocated to developing fruits, thereby accelerating sugar accumulation in fruits.

The transcription of genes related to polysaccharide degradation was increased (**Figure 7**), which contributed to the accelerated soluble sugar accumulation and berry softening observed in the G group. The enzymes associated with polysaccharide degradation such as amylases, endoglucanases, and pectin esterase produced disaccharides and monosaccharides, which constitute the soluble sugar in grape berries. In this study, a number of genes related to polysaccharide degradation showed significantly higher expression in the G group than in the CK group, in accordance with the increased soluble sugar content (**Figure 7F**). Pectin and cell wall degradation can lead to berry softening. PME1, which is an early marker for veraison (Barnavon et al., 2000), participated in pectin degradation and was more highly expressed in the G group than in the CK group (**Figure 3B**), suggesting that girdling promoted berry development.

In grape berries, sugars accumulate in the vacuoles of mesocarp cells, mainly in the form of glucose and fructose with a stable low level of sucrose (Lecourieux Ouaked et al., 2013). Sucrose in berries transported *via* the phloem is broken down into glucose and fructose by invertases, or into uridine-5′-diphosphate (UDP) glucose and fructose by SUS (Geigenberger and Stitt, 1993; Koch, 1996). These monosaccharides are transferred from related organelles to vacuoles by a hexose transporter (Hayes et al., 2007). Sucrose and hexose transporter genes are known to play vital roles in soluble sugar accumulation in berry vacuoles at the ripening stage (Davies et al., 1999; Fillion et al., 1999; Desvignes et al., 2005). However, expressions of these genes in berries did not differ significantly between the G and CK groups. Instead, the effect of girdling on sucrose metabolism in berries was observed at both the transcriptional and biochemical levels (**Figures 7E, F**). In addition to cleavage activity, SUS also catalyzes sucrose synthesis, which is the reverse reaction of sucrose cleavage. The net SUS activity was catalytic, resulting in increased sucrose synthesis (**Figure 7E**). Additionally, sucrose can be synthesized from glucose and fructose by SPS (Zhu et al., 2017). Thus, the downregulation of VIT217s0053g00700 (SUS) and VIT218s0089g00410 (SPS) expression in the G group may promote soluble sugar accumulation in berries by reducing sucrose synthesis activity.

Carbohydrates can be produced in green grape skin *via* photosynthesis, as green skin cells contain chlorophyll (Deytieux et al., 2007). From the beginning of veraison, chlorophyll content in grape skin cells gradually declines with berry development, which may be accompanied by the downregulation of genes involved in photosynthesis (Waters et al., 2005). In our study, most of the 72 DEGs involved in photosynthesis in berries were downregulated in the G group relative to the CK group, especially in G30, implying that girdling promoted grape berry development. The suppression of photosynthesis at the transcriptional level may be the result of negative feedback regulation of sugar accumulation in berries. Girdling of branches decreased photosynthetic electron fluxes and conferred sustained photoprotection against photodamage to mango leaves (Urban and Alphonsout, 2007). Similar protective mechanisms may also be in effect in developing grape berries.

Skin color changes markedly after the start of veraison in red grapes such as ‘Summer Black’. Anthocyanins, which mostly accumulate in skin cells, are responsible for the red coloration of grape berries (Fernández-López et al., 1998). Contrary to the change in chlorophyll content, anthocyanin content in skin cells started to increase from the beginning of veraison until full berry ripening (Champa et al., 2014). It was previously reported that anthocyanin content in the fruit skin of *Prunus cerasifera* var. Pissardii increased linearly with girdling duration (Lo Piccolo et al., 2021), which is supported by the observed increase in total anthocyanin content in berries (**Figure 5B**). The biosynthesis of anthocyanins involves a series of reactions that include phenylpropanoid and flavonoid biosynthesis (Shangguan et al., 2017). Key enzymes of the flavonoid biosynthesis pathway including PAL, CHS, and chalcone isomerase participate in anthocyanin biosynthesis (Ferri et al., 2011). Transcription of genes encoding these enzymes may be modulated by sucrose, and transcript levels are increased by higher exogenous sucrose content. We observed the upregulation of *PAL*, *CHS*, and chalcone isomerase genes mainly in G10 and G20, which may have resulted from the modulation of sucrose levels through vigorous phloem transport induced by trunk girdling. Thus, the upregulation of these three genes potentially contributed to anthocyanin accumulation.

In addition to the modulation of sucrose, transcription factors also participated in regulating the expression of genes involved in various physiologic processes such as anthocyanin biosynthesis. A number of MYB, basic helix–loop–helix (bHLH), and tryptophan–aspartic acid repeat (WDR) transcription factors in grapes control the expression of structural genes of the anthocyanin biosynthesis pathway (This et al., 2007; Hichri et al., 2010; Matus et al., 2010). For example, VvMYBAs promote VvUFGT (UDP-glucose:flavonoid 3-*O*-glucosyltransferase) expression and were found to positively regulate the later stages of anthocyanin synthesis, modification, and transport in cv. Shiraz (Rinaldo et al., 2015). VvMYBA1 is responsible for the loss of pigmentation in white-skinned grapes because the Gret1 retrotransposon inserts into the promoter region of *VvMYBA1* and disrupts its function of promoting VvUFGT activity (Kobayashi et al., 2004). VvMYBA3 was also reported to be truncated and possibly nonfunctional (Walker et al., 2007). Of the three studied VvMYBAs in grapes, VvMYBA2 plays a key role in upregulating the expression of *UFGT* genes and increasing anthocyanin accumulation in red grape berries (Niu et al., 2018). In this study, we did not detect the expression of *VvMYBA1*, and *VvMYBA2* and *VvMYBA3* transcript levels were significantly elevated in the G group (**Figure 8** and **Supplementary Table 6**). Indeed, the expression of VIT211s0052g01630 and VIT216s0039g02230, both encoding VvUFGTs, was significantly upregulated in G10 and G20, which is consistent with the upregulation of VIT202s0033g00390 (VvMYBA2; **Supplementary Table 6**). As VvUFGT is the final and key enzyme in the biosynthesis of anthocyanins, its increased expression may promote anthocyanin accumulation. However, only a few of the 120 differentially expressed transcription factors have been annotated.

Phytohormones including ABA, ETH, BR, JA, CK, GA, and auxins, which are also known as plant growth regulators, have been implicated in the control of grape berry development (Deluc et al., 2007). ABA, ETH, and BR promote grape ripening, whereas auxins such as IAA delay grape ripening (Kuhn et al., 2013). In our study, two *MetK* and two *ACCO* genes were upregulated under girdling relative to the control treatment (**Table 1**). MetK and ACCO are enzymes that participate in ETH biosynthesis; higher transcript levels of *MetK* and *ACCO* can lead to increased ETH production and release in berries under girdling, further increasing anthocyanin biosynthesis gene expression and total anthocyanin content (El-Kereamy et al., 2003). DEGs associated with the biosynthesis of ABA (except VIT205s0051g00670, NCED), BR, and IAA were significantly downregulated in the G group (**Table 1**). In terms of the IAA response, most DEGs encoding the auxin-responsive proteins IAA, ARF, GH3, and SUAR were downregulated in the G group compared with the CK group (**Table 2**). This may have delayed the corresponding processes, ultimately promoting berry ripening. One *PP2C* gene (ABA response) and 12 *TCH4* genes (BR response) were significantly upregulated by girdling (**Table 2**), demonstrating that this practice strongly promotes berry ripening at the level of gene transcription.

## Conclusion

**Figure 9:**
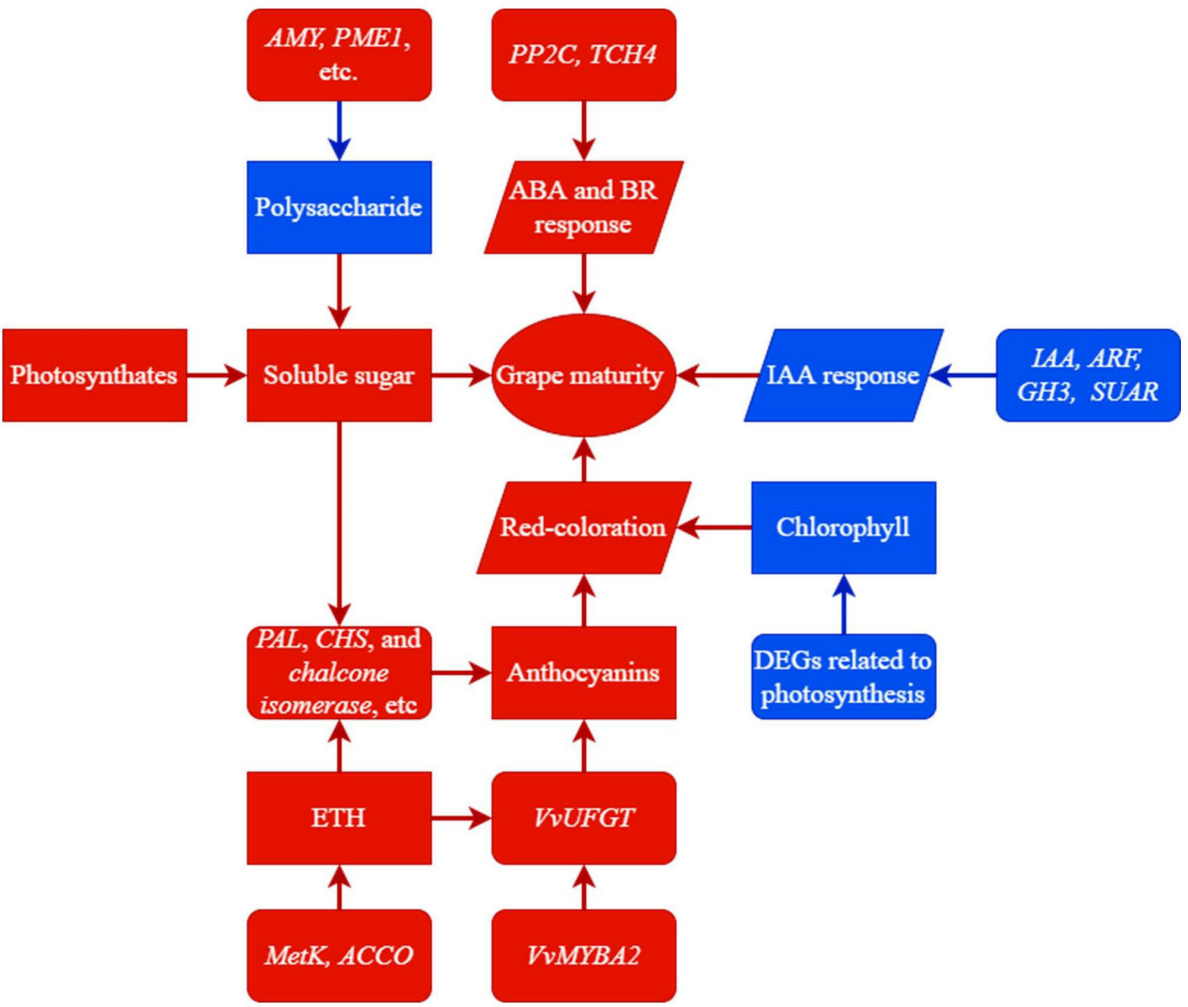
A summary of trunk girdling effects on berry development promotion. Rectangles represent contents of substances. Parallelograms represent activities of biological processes. Rounded rectangles represent expressions of genes. Red color in directional connectors represents a positive effect on terminal side; otherwise, it represents an increase in expression, content, or activity. Blue color in directional connectors represents a negative effect on terminal side; otherwise, it represents a decrease in expression, content, or activity.

In this study, we performed transcriptomic and physiologic analyses to investigate the molecular basis for the maturation-promoting effect of trunk girdling at early veraison in grape berries. Girdling promoted sugar accumulation and color change in berries and advanced berry ripening by 25 days (**Figure 9**). The accelerated sugar accumulation in the G group may have resulted from the reallocation of photosynthates to berry instead of root *via* phloem transport, as well as enhanced sucrose cleavage and polysaccharide degradation activities. Additionally, polysaccharide degradation may have resulted in berry softening. The acceleration of red coloration in the G group was caused by the upregulation of genes involved in chlorophyll degradation and anthocyanin accumulation. Most DEGs involved in anthocyanin biosynthesis were upregulated by girdling, and many were modulated by sucrose and transcription factors. For example, the expression of *VvUFGT*, a key gene in anthocyanin biosynthesis, increased in association with *VvMYBA2* expression in berries of the G group. Girdling also enhanced the expression of genes encoding MetK, ACCO, PP2C, and TCH4, which enhanced the ripening-promoting effects of ETH, ABA, and BR. In the G group, most DEGs encoding the auxin-responsive proteins IAA, ARF, GH3, and SUAR were downregulated relative to the control, which may have reduced the IAA response and mitigated the delaying effect of IAA on berry development and ripening. In total, 120 transcription factors were differentially expressed under girdling treatment, which may play important roles in regulating berry development and ripening. Our findings provide a global view of the molecular changes induced by trunk girdling at early veraison in grapes and the positive effects of this practice on berry development. However, the function of many of the identified differentially expressed transcription factor genes is unknown, and further work is needed to determine their contribution to grape berry development and ripening.

## Data availability statement

The RNA-seq data presented in the study are deposited in the NCBI sequence read archive repository, accession number PRJNA866294.

## Author contributions

YoZ and YiZ conceived and designed the study. YP, XG and YoZ carried out the experiments and drafted the manuscript. YP, XG and JH analyzed the RNA sequencing data. QZ, YiZ, and ZL analyzed physiological data. All authors contributed to the article and approved the submitted version.

## Funding

This research was supported by the Research Foundation of Leshan Normal University for Talented Scholars (No. RC202005), Academic Discipline Breeding Project of Leshan Normal University (2022SSDJS007) and Scientific And Technological Projects of Sichuan Province (2020YJ0351).

## Acknowledgments

The authors are grateful to the online platform of Majorbio Cloud Platform (www.majorbio.com) for transcriptional data analysis. The authors are also grateful to Doctor Tao Meng (Leshan Normal University, Leshan, China) for the help in the improvement of the photos in **Figure 1**.

## Conflict of interest

The authors declare that the research was conducted in the absence of any commercial or financial relationships that could be construed as a potential conflict of interest.

## Publisher’s note

All claims expressed in this article are solely those of the authors and do not necessarily represent those of their affiliated organizations, or those of the publisher, the editors and the reviewers. Any product that may be evaluated in this article, or claim that may be made by its manufacturer, is not guaranteed or endorsed by the publisher.
